# A relaxation technique enhances psychological well-being and immune parameters in elderly people from a nursing home: A randomized controlled study

**DOI:** 10.1186/1472-6882-14-311

**Published:** 2014-08-23

**Authors:** Abilio Reig-Ferrer, Rosario Ferrer-Cascales, Ana Santos-Ruiz, Adolfo Campos-Ferrer, Alvaro Prieto-Seva, Irene Velasco-Ruiz, Maria Dolores Fernandez-Pascual, Natalia Albaladejo-Blazquez

**Affiliations:** Department of Health Psychology, Faculty of Health Sciences, University of Alicante, Alicante, Spain; Immunohematology Service, Transfusion Centre of Alicante, Department of Clinical Medicine, University of Miguel Hernandez, Alicante, Spain; Consellería de Bienestar Social. Generalitat Valenciana, Alicante, Spain; University Hospital of San Juan, Obstetrics and Gynecology Service, Department of Biotechnology, University of Alicante, Alicante, Spain

**Keywords:** Elderly, Immune system, Meditation, Nursing homes, Quality of life, Relaxation response, Tranquilization

## Abstract

**Background:**

The aging process involves a decline in immune functioning that renders elderly people more vulnerable to disease. In residential programs for the aged, it is vital to diminish their risk of disease, promote their independence, and augment their psychological well-being and quality of life.

**Methods:**

We performed a randomized controlled study, evaluating the ability of a relaxation technique based on Benson’s relaxation response to enhance psychological well-being and modulate the immune parameters of elderly people living in a geriatric residence when compared to a waitlist control group. The study included a 2-week intervention period and a 3-month follow-up period. The main outcome variables were psychological well-being and quality of life, biomedical variables, immune changes from the pre-treatment to post-treatment and follow-up periods.

**Results:**

Our findings reveal significant differences between the experimental and control groups in CD19, CD71, CD97, CD134, and CD137 lymphocyte subpopulations at the end of treatment. Furthermore, there was a decrease in negative affect, psychological discomfort, and symptom perception in the treatment group, which increased participants’ quality of life scores at the three-month follow-up.

**Conclusions:**

This study represents a first approach to the application of a passive relaxation technique in residential programs for the elderly. The method appears to be effective in enhancing psychological well-being and modulating immune activity in a group of elderly people. This relaxation technique could be considered an option for achieving health benefits with a low cost for residential programs, but further studies using this technique in larger samples of older people are needed to confirm the trends observed in the present study.

**Trial registration:**

International Standard Randomised Controlled Trial Number Register ISRCTN85410212

## Background

As we age, our immune response declines and there is a greater susceptibility to disease due to a reduced ability to fight infection or to accurately recognize foreign agents or agents from the organism itself [1, 2]. Various authors have defended the working hypothesis that certain psychological interventions are able not only to augment an individual’s psychological well-being, but also to improve cell immune competence in humans [3–5]. Scientific evidence, with respect to the utility and efficacy of various psychological treatments and their beneficial effects, is positive and solid [6–8], but the potential benefits of psychological interventions on immune modulation is much less noteworthy. The significant clinical implications of the hypothetical relationship between psychological treatment and immune modulation has steered an ample number of empirical studies that aim to study such relationship in various population samples [9, 10]. For instance, a psychological program for stress management was able to modify the immune response and the course of the disease in patients with skin cancer [3, 4, 11]. A cognitive-behavioral therapy approach yielded similar results by leading to increased production of interleukins in patients with breast cancer during the six months following psychological treatment [12]. However, contrasting results were found in women with autoimmune disease for whom cognitive-behavioral therapy changed emotional variables and enhanced quality of life without altering immunological parameters [13]. In recent years, interest in optimizing immune function has led to investigating the efficacy of alternative techniques in modulating immune system activity in healthy individuals, such as the practice of Hatha yoga in normal adults [14], or of aerobic exercise in elderly people [15]. A recent study showed that a deactivation technique (i.e., mindfulness-based stress reduction) downregulated the expression of the NF-кB pro-inflammatory gene in older adults [16]. Using a different approach, Kiecolt-Glaser et al. [5] reported that training in a Jacobson-type muscle relaxation technique (tensing and relaxing muscle groups) enhanced cellular immune competence in the elderly.

Such optimization of immune function in older people would have a great impact on their health status, decreasing their susceptibility to disease and enhancing their quality of life. Empirical evidence shows that our immune systems weaken as we age and that factors such as genetics or chronic stress could accelerate immune decline [17, 18].

Although Jacobson’s relaxation technique effectively enhanced cellular immune competence in older people [5], the increased prevalence of muscle and joint disorders in the elderly could hinder training and continued practice of the technique. Consequently, we decided to replace an active relaxation technique requiring muscle engagement with an alternative, largely passive technique that did not entail muscle relaxation and contraction. Benson’s relaxation response [19] fit the requirements and has proven efficacious in decreasing stress [20] and anxiety, as well as in enhancing cognitive performance in healthy aging [21]. In this study, we chose to label the intervention as a “tranquilization technique” because this term is well received by the elderly and it has a calming psychological effect in the target population.

It is worth noting that older people are capable of learning relaxation and meditation techniques adequately. These methodologies have proven effective in decreasing feelings of anxiety and hopelessness [22]. Some authors suggest that relaxation, meditation, and guided imagery (e.g., positive visualization) techniques are appropriate in working with components of spiritual health that permit a greater perception of well-being, a better connection with the self, with others, and with a higher power [23]. Therefore, the aim of this study was to test the effects of a psychological program of geriatric intervention based on the application of a passive relaxation technique, namely Benson’s relaxation technique, on psychological well-being and immune parameters in elderly people residing in a nursing home. We hypothesized that the Benson’s technique would generate positive effects on clinical and psychological well-being.

## Methods

### Participants

Participants were 30 residents of a nursing home (79% women), with a mean age of 83 years (SD = 4.97) and ranging between 74 and 91 years. They all were briefed on the details of the study and the intervention it entailed by the physician of the institution. Those individuals interested in participating that fulfilled inclusion criteria were admitted into the study.

The following were the inclusion criteria: (1) not to be taking drugs with significant effects on endocrine or immune function (e.g., corticosteroids or antimetabolites); (2) not to have health problems directly related to immune function (i.e., endocrine diseases, cancer, arthritis, asthma, or infectious diseases); and (3) not have endured an extremely negative life event within the year prior to study participation. The exclusion criterion was that there were indications of cognitive impairment. All participants gave their informed consent to the study, which had been approved by the Ethics Committee of the nursing home and of the University of Alicante, and followed the guidelines of the Helsinki Declaration (AMM, 2008) and the Good Clinical Practice Directive (Directive 2005/28/EC) of the European Union.

Participants were randomly assigned to one of two groups, by means of SPSS statistical software (version 20), with process of random numbers: the treatment group (TG; n = 15) and a control group that was waitlisted for intervention (CG; n = 15). The physician generated the random allocation sequence, enrolled participants and assigned participants to each groups. All participants underwent medical and clinical tests, measures of psychological well-being and of health-related quality of life.

Functional assessment of the elderly was made by means of the Katz Index of Activities of Daily Living (ADL) [24], Spanish adaptation by Alvarez et al. [25]. Most participants were autonomous in their daily living activities (i.e., bathing, dressing, toilet hygiene, functional mobility, bowel and bladder control, and feeding). Exceptions to functional autonomy were three older individuals from the control group (B Index) and one from the treatment group (C Index). There were no changes in functional status throughout the intervention, except for one member of the treatment group who suffered a transient ischemic attack (TIA) and went from a Katz Index A to a Katz Index G (i.e., dependent for all Activities of Daily Living).

### Measures

#### Psychological assessment

Psychological well-being and quality of life were assessed by means of tests with adequate psychometric properties that were culturally adapted to the sample. All instruments were administered by appropriately trained personnel.

##### Satisfaction with Life Scale (SWLS)

This scale consists of five statements measuring degree of life satisfaction in a Likert-scale format that goes from ‘strongly agrees’ to ‘strongly disagrees’ [26, 27]. SWLS’ validity and reliability are satisfactory [26]. Internal consistency of the scale, as measured by Cronbach’s alpha, is greater than 0.80 in the elderly population [28, 29], and the average coefficient for different populations is 0.75 [30]. The SWLS evaluates life satisfaction as current and past assessments of the degree to which individuals have attained their desired or planned life goals. Consequently, life satisfaction as measured by this instrument provides a reflective and dispassionate evaluation of how well things are going or have gone up to the present. We have used the Spanish adaptation of Arce’s [31], which we have successfully applied in previous research [27].

##### Affect Balance Scale (ABS)

Bradburn’s *Affect Balance Scale* [32] is aimed at determining a person’s psychological well-being at any given time. In the present study, we used a Spanish adaptation for elderly people by Stock et al. [33]. Psychometric characteristics of the scale are adequate, with the authors reporting an internal consistency of affect balance of 0.85, and a Cronbach’s alpha of 0.74 in a sample of Canadian elderly [34]. It appears, therefore, that the ABS is a sound measure of state affective well-being. It is administered by an interviewer and includes 10 questions that have two possible answers: ‘yes’ (1) or ‘no’ (0). Five of the questions correspond to positive affect, while the remaining five are related to negative affective states. The *affect balance* score is calculated by adding the positive items (A, C, E, G, I), dividing them into the sum of the negative items (B, D, F, H, J), and adding 5 points to the resulting number. The score ranges from 0 to 10, from more negative to more positive affect. The *hedonic balance* results from subtracting the sum of the negative items from that of the positive items, with an average value of zero and a range between -5 and +5. Positive scores correspond to a positive balance or euthymic tone, whereas negative scores indicate a predominance of dysthymic tone.

##### Nottingham Health Profile (NHP)

The *Nottingham Health Profile* [35] is a questionnaire that evaluates health-related quality of life. We used a Spanish adaptation for elderly people by Richart-Martinez et al. [29]. It contains two sections, with the first one measuring the perceived health status in terms of a series of regular problems or complaints that people have in their daily lives, while the second section focuses on activities that can be affected by the health status of the individual. The first section includes 38 questions pertaining to six areas: Energy, Pain, Emotional Reactions, Sleep, Social Isolation, and Physical Mobility. Interviewees answer ‘yes’ or ‘no’ to each question. The second section consists of ‘yes’ or ‘no’ statements about seven areas of life that are most affected by health status.

This instrument was selected because it is one of the most frequently used with geriatric populations, is easy to understand, and is readily accepted by elderly people [29, 35, 36]. Furthermore, it has acceptable psychometric properties [29, 36], with test-retest reliability over 0.70 for each of the scales in the first portion of the questionnaire, and between 0.44-0.86 for items in the second portion of the profile, in addition to good content and criterion validity [29, 35].

##### Benson’s Symptom List

Benson suggested that practicing the relaxation technique had beneficial effects on a series of symptoms: anxiety and hyperventilation; insomnia; headaches; back pain; and thoracic pain [37]. Global scores of the Benson Symptom List range from 1 to 5 points.

#### Assessment of biomedical variables

The following measures were taken at the beginning of treatment, 15 days following the end of the treatment sessions, and at follow-up, three months after finishing treatment:

- Clinical history from the start of the treatment program and for the following 6 months. The presence of multiple pathologies, which characterize geriatric populations, intercurrent diseases, and pharmacological treatments were registered throughout the duration of the study.
- Functional assessment by means of Katz Index of ADL [25].
- Physical parameters: Weight, height, Body Mass Index (BMI), blood pressure, and heart rate.
- Hematological parameters: red and white blood cells counts, and blood differential (CBC), hemoglobin, hematocryte, mean corpuscular volume (MCV), platelets, and erythrocyte sedimentation rate (ESR).
- Blood biochemistry measures: Glucose, creatinine, urea, uric acid, total cholesterol, HDL and LDL cholesterol, triglycerids, AST, ALT, GGT, total proteins, and serum albumin.

Hematologic and biochemical parameters were assayed in the laboratory of the Service of Clinical Analysis of the San Juan Clinical University Hospital in Alicante (Spain). Blood samples were collected in the morning following a 12-hour fast. Samples were processed immediately. At that same time, blood samples collected to assess immune function were refrigerated and sent to the laboratory within 6 hours after collection. Research staff taking measurements and processing samples were blinded to the group condition (treatment or waitlist group).

#### Immunological assessment

Immune cells carry out some of their functions by means of cell-to-cell contact and via membrane receptors or antigens that are expressed at the cellular surface. Some molecules appear at various stages of cell differentiation or activation. In the present study, we recorded the following molecules: CD4, CD8, CD19, CD56, CD71, CD97, CD134, and CD173.

Phenotypic analysis of the various cell populations was carried out by immunofluorescence by means of flow cytometry with double or triple labeling. This technique constitutes a useful tool for cell identification and characterization. Following intravenous peripheral blood collection, 50 μl of blood were incubated for 10 minutes with 3 μl of the following combinations of monoclonal antibodies (Pharmigen):

- CD4-PE + CD8-FITC + CD19-Cy5
- CD8-FITC + CD56-PE
- CD71-FITC + CD97-PE
- CD134-FITC + CDw137-PE

Afterwards, 1 mL of a hematolysis solution (Quicklysis, Cytognos) was added and the blood solution was kept in the dark for 5 minutes, after which time 10,000 cells were subjected to cytometric analysis.

Immunofluorescence analysis was carried out by means of a Vantage (Becton Dickinson) FACS flow cytometer, equipped with an argon laser (488 nm) that excites the FITC, PE, and Cy5 fluorchromes, emitting at 520, 575, and 667 nm, respectively.

### Psycho-gerontology intervention

Psychological treatment was conducted by an expert psychologist in the practice of this technique. It consisted of empirical training in the “relaxation response” technique designed by Benson [19], which we named the “tranquilization technique”. The technique is extremely effective in controlling and decreasing stress levels and the associated tension, as well as in bringing about a sense of personal well-being. The following requirements are of paramount importance: (a) a quiet environment; (b) a word or phrase that is repeated and on which the person focuses attention; (c) a passive attitude; and (d) a comfortable posture.

This relaxation technique consists of engaging in repeated resting periods in which the mind is free from preoccupations and the body is liberated of all tension. This state is reached by focusing on a special object, usually a word o phrase. In this study, the word “dos” (“two” in English) was used to achieve the relaxation response.

### Procedure

The study was conducted in a relaxing and quiet room of a public nursing home in Alicante, Spain. Chairs were mobile and comfortable.

On the first day, the researchers carrying out the study introduced themselves and the task to be accomplished. They explained the aims of the relaxation technique (i.e., to enhance their physical and psychological well-being, and to achieve a greater personal serenity), the reason for the questions they would be asked immediately (pre-test), as well as at the end of the relaxation training, and three months after the study was finished. Researchers also explained the purpose of the various analyses and clinical tests, and the need to have them repeated.

Several questionnaires and tests (see 2.2. Psychological assessment) were individually administered on the first and last days of treatment. On those occasions, participants were also asked about the symptoms and health problems that Benson claimed could be modified by practicing his relaxation technique: anxiety and hyperventilation, insomnia, headaches, back pain, thoracic pain, hypertension, heart rate and heart problems, secondary symptoms of cancer, and cholesterol levels.

Hour-long group sessions were conducted daily, from Monday through Friday, for a total of 10 days in a span of two weeks. At the end of each group session, participants were encouraged to practice the technique two or three more times within the 24 hours leading to the next session. Training sessions were scheduled at noon, a time that was several hours away from breakfast and the main meal of the day (in Spain).

Assessments were repeated at the end of treatment and three months later. On this last evaluation, the treatment group was asked for a subjective appraisal of the relaxation experience and their practice routine over the three months leading to the follow up.

The control group (i.e., the waitlist group) was assessed in the same fashion as the treatment group and following the same sequence (right before the beginning of the treatment, at the end, and three months later).

### Statistical analysis

Type of treatment (relaxation technique vs. waitlist) divided participants into two groups: treatment and control. All other variables were considered repeated measures: tests and questionnaires about psychological well-being and quality of life, clinical and biomedical tests, and immunological tests.

All statistical analyses were conducted by means of SPSS statistical software (version 20). The non-parametric Mann–Whitney *U* test for independent samples was used to test for group effects on the various dependent measures at the three assessment times (A = before treatment; B = immediately after treatment; C = three months after treatment).

Within-subject pre- and post-treatment effects on the dependent measures compared pre-treatment to post-treatment (A vs. B), and pre-treatment to the three-month follow-up (A vs. C). It was performed by means of Wilcoxon’s *T* test, a non-parametric test for related samples.

## Results

The nursing home had 100 residents, and 70 were excluded for not meeting inclusion criteria or because they declined participation in the study. Thirty participants were randomly assigned to each group. The treatment group consisted of 15 people, but just 11 participants finished the intervention, because two participants never initiated the treatment after randomization, one participant had to be hospitalized due to a diabetic nephropathy that caused his death; a second one suffered a TIA that resulted in hemiplegia and aphasia. The waitlist group, consisted of 15 people in the beginning, but one participant was not evaluated at the follow-up phase due to a TIA with hemiplegia and aphasia.

**Figure 1:**
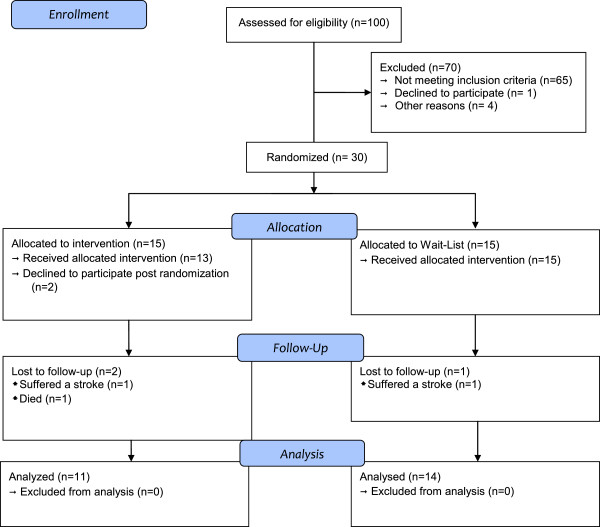
**CONSORT flowchart of participants retained at each phase of the trial.**

Figure 1 includes a CONSORT flowchart, detailing the recruitment procedure, group assignment, and reasons for attrition for each group of participants.

Below, we report the most significant results, grouped into psychological, clinical-medical, and immunological variables. Each subheading includes data for both groups (treatment vs. waitlist control) and three assessments (before treatment, immediately after treatment, and three-month follow up).

### Psychological assessment

Table 1 includes results of psychological tests for both groups. Pre-treatment (time A) testing shows that psychological well-being was reasonably good for both groups, taking into account that all participants were elderly and residing in a nursing home. Results indicate that participants had levels of psychological well-being (i.e., mood and life satisfaction) comparable to those of elderly people in a community setting.

**Table 1:** **Means (M) and standard deviations (SD) of psychological measures for participants of both groups**

Variable	Group	A M ± SD	Mann-Whitney*U*		B M ± SD	Mann-Whitney*U*		C M ± SD	Mann-Whitney*U*		Wilcoxon’s*T*A vs. B		Wilcoxon’s*T*A vs. C	
			*z*	*p*		*z*	*p*		*z*	*p*	*z*	*p*	*z*	*p*
*SWLS*	TG	16.46 ± 5.24	-0.39	0.69	17.92 ± 3.71	-0.34	0.72	16.91 ± 4.74	-1.07	0.28	-1.53	0.12	0.00	1.00
	CG	17.80 ± 3.34			17.13 ± 5.17			18.93 ± 3.84			-0.51	0.60	-0.94	0.34
Positive Affect	TG	2.85 ± 1.34	-1.85	0.06	2.54 ± 0.97	-0.19	0.84	2.73 ± 1.10	-0.62	0.53	-0.78	0.43	-0.99	0.31
	CG	2.00 ± 1.11			2.40 ± 1.24			2.40 ± 1.24			-1.22	0.22	-1.23	0.21
Negative Affect	TG	3.15 ± 1.21	-1.59	0.11	2.38 ± 0.96	-0.55	0.58	3.00 ± 1.34	-0.80	0.42	-1.89	**0.05**	-0.36	0.71
	CG	2.36 ± 1.34			2.60 ± 1.12			2.60 ± 1.24			-0.18	0.85	-0.36	0.71
Affect:	TG	-0.31 ± 1.60	-0.47	0.63	0.15 ± 0.69	-0.83	0.40	-0.27 ± 0.90	-0.29	0.76	-0.99	0.32	-0.66	0.50
Hedonic balance	CG	-0.36 ± 1.39			-0.20 ± 1.21			-0.20 ± 1.08			-0.85	0.39	-0.55	0.58
Affect:	TG	4.69 ± 1.60	-0.47	0.63	5.15 ± 0.69	-0.83	0.40	4.73 ± 0.90	-0.29	0.76	-0.99	0.32	-0.66	0.50
Standardized ABS	CG	4.64 ± 1.39			4.80 ± 1.21			4.80 ± 1.08			-0.08	0.39	-0.55	0.58
*NHP*														
Energy	TG	1.77 ± 1.17	-0.40	0.68	2.00 ± 1.15	-0.92	0.35	1.36 ± 1.43	-0.51	0.60	-0.87	0.38	-0.96	0.33
	CG	1.53 ± 1.30			1.60 ± 1.12			1.53 ± 1.19			-0.44	0.65	0.00	1.00
Pain	TG	3.62 ± 2.90	-1.00	0.31	3.46 ± 3.10	-0.67	0.49	2.91 ± 2.51	-0.13	0.89	-0.10	0.91	-1.02	0.30
	CG	2.60 ± 2.64			2.87 ± 3.27			3.00 ± 3.16			-0.27	0.78	-0.49	0.62
Emotional	TG	4.85 ± 1.68	-2.01	**0.04**	3.54 ± 1.85	-0.02	0.98	3.64 ± 2.98	-0.18	0.85	-2.22	**0.02**	-1.79	0.07
Reactions	CG	3.53 ± 1.85			3.73 ± 2.31			3.13 ± 1.88			-0.14	0.88	-0.71	0.47
Sleep	TG	2.54 ± 1.56	-0.46	0.63	2.46 ± 1.61	-0.12	0.90	3.09 ± 1.30	-1.36	0.17	-0.58	0.56	-1.41	0.15
	CG	2.27 ± 1.67			2.53 ± 1.85			2.13 ± 1.92			-1.15	0.24	-0.43	0.66
Social	TG	1.23 ± 1.24	-0.26	0.79	0.85 ± 0.99	-0.53	0.59	0.91 ± 1.22	-0.08	0.93	-0.50	0.13	-1.29	0.19
Isolation	CG	1.33 ± 1.23			1.27 ± 1.49			1.13 ± 1.46			-0.06	0.95	-0.88	0.37
Physical	TG	4.15 ± 2.12	-0.81	0.41	4.31 ± 2.81	-1.06	0.28	3.36 ± 2.46	-0.07	0.93	-0.41	0.68	-1.93	**0.05**
Mobility	CG	3.67 ± 2.29			3.13 ± 2.67			3.33 ± 2.19			-1.24	0.21	-0.81	0.41
NHP Total	TG	18.15 ± 7.49	-1.08	0.27	16.62 ± 8.33	-0.43	0.66	15.27 ± 7.88	-0.39	0.69	-1.02	0.30	-1.89	**0.05**
	CG	14.93 ± 6.93			15.13 ± 9.78			14.27 ± 9.07			-0.11	0.90	-0.66	0.50
Benson’s Five	TG	2.85 ± 1.28	-1.18	0.23	1.83 ± 1.03	-0.40	0.68	2.36 ± 1.43	-0.79	0.42	-2.04	**0.04**	-0.95	0.34
Symptoms	CG	2.27 ± 1.53			2.00 ± 1.20			1.87 ± 1.51			-0.81	0.41	-1.11	0.26

Note: Between-group comparisons (Mann–Whitney *U* test) at three assessment times; and within-subject analyses (Wilcoxon’s *T*) comparing pre-treatment assessment (A) with post-treatment assessment (B) and follow-up assessment (C). TG: Treatment Group; CG: Waitlist Control Group; M: Mean (SD: Standard Deviation); ABS: Affect Balance Scale; SWLS: Satisfaction with Life Scale; NHP: Nottingham Health Profile.

Bold text: *p* < 0.05.

Statistical analyses also demonstrated that there were no significant differences between groups prior to relaxation training. The single exception to this pattern was the Emotional Reactions of the Nottingham Health Profile, whereby scores were higher for the treatment group. These differences, however, disappeared over the course of the following evaluations, with the treatment group reporting less psychological distress after treatment (*p* = 0.02), in comparison with the control group, whereby no differences are observed.

Similarly, negative affect scores of the *Affect Balance Scale* decreased after treatment for the treatment group, with results being marginally significant (*p* = 0.05). The relaxation technique had a positive impact on self-report symptom measures (*p* = 0.04), with the treatment group reporting fewer symptoms after treatment. All other psychological variables remained unaffected by the intervention at the various assessment times.

The waitlist control group did not show any changes in any of the measures of psychological well-being or quality of life at any of the assessment points.

**Figure 2:**
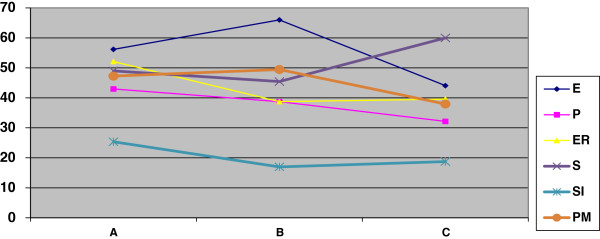
**Weighted scores for the treatment group: Nottingham Health Profile at the three assessment times.** A: pre-treatment assessment; B: post-treatment assessment; C: three-month follow-up assessment. E: Energy; P: Pain; ER: Emotional Reactions; S: Sleep; SI: Social Isolation; PM: Physical Mobility.

Results indicate a trend toward improvement of health-related quality of life scores following treatment (NHP total score, *p* = 0.05). This trend suggests that health-related quality of life continues to improve over time after relaxation training (A = 18.15, B = 16.62, and C = 15.27; whereby lower scores represent better outcomes), although the trend does not reach statistical significance. Figure 2 includes weighted averages for each of the NHP scales at each of the three assessment times, showing how all scale scores decreased over time, with the exception of the scale measuring sleep problems.

### Biomedical and clinical measures

#### Description of clinical variables for all participants

Table 2 includes all prevalent pathologies in the treatment and waitlist control groups.

**Table 2:** **Prevalence of medical pathologies**

	Treatment group	Control group
Hypertension	8	8
Mood disorders	7	4
Sensory disorders	5	-
Musculoskeletal pathologies	4	4
Diabetes	3	3
Heart failure	2	2
COPD	1	-
Benign prostatic hyperplasia	1	-
Parkinson’s disease	1	-
Acute myocardial infarction	-	1
Pacemaker	-	2
Chronic renal failure	-	2
Previous stroke	-	1
Adrenal/thyroid insufficiency	-	1

Note: COPD: Chronic Obstructive Pulmonary Disease.

#### Medical and laboratory measures

Table 3 presents all clinical-biomedical results for both groups at the three assessment times.

**Table 3:** **Clinical measures of both groups at three assessment times**

Variable	Group	A M ± SD	Mann-Whitney*U*		B M ± SD	Mann-Whitney*U*		C M ± SD	Mann-Whitney*U*		Wilcoxon’s*T*A versus B		Wilcoxon’s*T*A versus C	
			*z*	*p*		*z*	*p*		*z*	*p*	*z*	*p*	*z*	*p*
SBP (mmHg)	TG	143.08 ± 21.65	0.00	1.00	138.08 ± 19.53	-0.74	0.45	135.00 ± 19.16	-0.66	0.51	-0.90	0.36	-1.63	0.10
	CG	141.67 ± 19.88			144.44 ± 20.53			141.67 ± 17.22			-0.77	0.43	-0.63	0.52
DBP (mmHg)	TG	75.77 ± 12.72	-0.37	0.70	74.62 ± 9.00	-0.27	0.78	67.50 ± 9.57	-1.26	0.20	-0.27	0.78	-1.13	0.25
	CG	72.33 ± 10.33			75.56 ± 9.82			75.00 ± 8.37			-0.43	0.66	-0.13	0.89
HR (bpm)	TG	79.08 ± 12.12	-0.95	0.34	80.09 ± 14.02	-1.07	0.28	-	-	-	-0.10	0.91	-	-
	CG	73.27 ± 8.45			73.00 ± 7.35			-			-0.35	0.72	-	-
Leukocytes/mm^3^	TG	6646.15 ± 1145.73	-0.29	0.77	6584.62 ± 1003.20	-1.23	0.21	6180.00 ± 1139.98	-0.78	0.43	-0.52	0.60	-1.63	0.10
	CG	6450.00 ± 1399.31			6192.31 ± 1515.18			6861.54 ± 1526.18			-0.35	0.72	-0.39	0.69
Segmented (%)	TG	56.74 ± 7.70	-0.34	**0.01**	56.38 ± 7.41	-2.30	**0.02**	59.44 ± 7.77	-1.67	0.09	-0.17	0.86	-1.07	0.28
	CG	47.55 ± 11.31			48.93 ± 8.56			51.89 ± 10.34			-1.05	0.28	-1.29	0.19
Limphocytes (%)	TG	30.55 ± 7.90	-1.82	0.06	30.66 ± 6.77	-2.38	**0.01**	28.92 ± 6.80	-1.36	0.17	-0.14	0.88	-0.81	0.41
	CG	38.02 ± 12.50			36.99 ± 7.16			35.48 ± 10.73			-0.15	0.87	-0.31	0.75
Monocytes (%)	TG	8.63 ± 1.63	-0.87	0.38	8.72 ± 1.92	-0.61	0.53	9.12 ± 2.02	-0.50	0.62	0.00	1.00	-1.71	0.08
	CG	9.67 ± 2.63			9.07 ± 1.99			8.72 ± 2.34			-0.27	0.78	-1.29	0.19
Eosinophils (%)	TG	3.56 ± 1.82	-0.72	0.46	3.64 ± 2.00	-0.64	0.52	3.64 ± 2.24	-0.59	0.55	0.00	1.00	-0.25	0.79
	CG	4.15 ± 2.60			4.28 ± 2.61			3.96 ± 1.99			-0.23	0.81	-1.11	0.26
Basophils (%)	TG	0.50 ± 0.21	-0.71	0.47	0.73 ± 0.58	-0.31	0.75	0.48 ± 0.12	-2.01	**0.04**	-1.07	0.28	-0.81	0.41
	CG	0.58 ± 0.33			0.70 ± 0.43			0.69 ± 0.33			-0.27	0.78	-1.54	0.12
Red blood (cells/mm^3^)	TG	4.42 ± 0.33	-1.05	0.29	4.49 ± 0.37	-1.13	0.25	4.53 ± 0.38	-1.34	0.17	-0.80	0.42	-1.68	0.09
	CG	4.30 ± 0.39			4.36 ± 0.49			4.31 ± 0.35			-1.29	0.19	-0.39	0.69
Hemoglobin (g/dl)	TG	13.47 ± 1.18	-1.43	0.15	13.61 ± 1.31	-1.07	0.28	13.90 ± 1.20	-1.40	0.16	-0.87	0.38	-2.19	**0.02**
	CG	12.86 ± 1.28			13.04 ± 1.67			13.08 ± 1.17			-0.93	0.35	-1.33	0.18
Hematocrit (%)	TG	41.66 ± 3.57	-1.67	**0.09**	41.75 ± 3.83	-1.18	0.23	41.52 ± 3.81	-0.59	0.55	-0.24	0.80	-0.15	0.87
	CG	39.35 ± 3.71			39.82 ± 4.31			40.57 ± 3.42			-0.90	0.36	-1.41	0.15
MCV (fl)	TG	94.23 ± 4.53	-1.62	0.10	93.40 ± 4.61	-1.10	0.27	92.14 ± 4.41	-1.18	0.23	-2.62	**0.00**	-2.49	**0.01**
	CG	91.45 ± 5.27			91.80 ± 5.14			94.29 ± 6.01			-0.49	0.62	-1.86	0.06
Platelets/mm^3^	TG	232.38 ± 49.53	-1.57	0.11	234.77 ± 55.25	-1.71	0.08	196.70 ± 37.78	-0.12	0.90	-0.38	0.70	-2.14	**0.03**
	CG	197.57 ± 41.64			197.00 ± 38.20			203.66 ± 62.30			-0.66	0.50	-0.15	0.87
ESR (mm/h)	TG	40.25 ± 21.93	-0.69	0.48	44.55 ± 31.70	0.22	0.82	29.14 ± 18.84	-0.76	0.44	-0.77	0.44	-0.94	0.34
	CG	35.36 ± 27.54			45.29 ± 23.96			41.50 ± 29.78			-0.63	0.52	-0.44	0.65
Glycemia (mg/dl)	TG	108.08 ± 23.46	-2.21	0.22	108.82 ± 31.59	-0.60	0.54	102.0 ± 29.21	-1.05	0.29	-0.89	0.37	-0.25	0.79
	CG	91.53 ± 15.08			103.69 ± 47.89			86.54 ± 19.33			-0.15	0.87	-1.54	0.12
Creatinine (mg/dl)	TG	0.92 ± 0.18	-0.87	0.38	0.92 ± 0.22	-1.45	0.14	0.97 ± 0.19	-1.68	0.09	-0.71	0.47	-2.00	**0.04**
	CG	1.01 ± 0.30			0.99 ± 0.18			1.13 ± 0.36			-1.45	0.14	-2.74	**0.00**
Urea (mg/dl)	TG	34.23 ± 9.75	-1.91	**0.05**	38.27 ± 6.39	-1.62	0.10	41.09 ± 8.70	-0.87	0.38	-2.32	0.02	-2.18	**0.02**
	CG	6.87 ± 20.27			46.31 ± 12.43			51.46 ± 20.53			-0.17	0.86	-1.18	0.23
Uric Acid (mg/dl)	TG	4.58 ± 1.23	-1.45	0.14	4.50 ± 1.55	-1.59	0.11	4.75 ± 1.41	-1.30	0.19	-1.11	0.26	-0.89	0.37
	CG	5.58 ± 1.81			5.52 ± 1.40			5.57 ± 1.50			-0.24	0.80	-0.17	0.86
Cholesterol (mg/dl)	TG	196.46 ± 29.01	-0.53	0.59	190.00 ± 35.79	-1.04	0.29	215.64 ± 40.38	-0.98	0.32	-0.22	0.82	-1.86	0.06
	CG	202.93 ± 46.63			210.54 ± 47.76			202.15 ± 30.02			-1.51	0.13	-0.35	0.72
HDL Cholesterol	TG	51.50 ± 12.66	-0.29	0.77	52.03 ± 13.29	-0.23	0.81	55.19 ± 13.74	-0.46	0.64	-0.10	0.91	-1.47	0.13
(mg/dl)	CG	53.00 ± 11.67			50.95 ± 9.66			56.35 ± 13.10			-1.12	0.26	-2.66	**0.00**
Triglycerides (mg/dl)	TG	135.46 ± 70.04	-0.73	0.46	128.18 ± 68.05	-0.40	0.68	137.36 ± 92.12	-0.20	0.83	-1.24	0.21	-0.35	0.72
	CG	126.20 ± 75.86			146.62 ± 95.11			104.00 ± 27.91			-0.98	0.32	-0.07	0.93
LDL Cholesterol	TG	117.86 ± 29.38	-0.68	0.49	112.32 ± 31.70	-1.04	0.29	131.47 ± 33.46	-0.97	0.33	0.00	1.00	-1.48	0.13
(mg/dl)	CG	130.77 ± 28.01			128.33 ± 36.95			125.32 ± 25.37			-0.84	0.40	-0.65	0.51
AST (U/L)	TG	22.37 ± 5.08	-0.76	0.44	19.88 ± 5.97	-0.66	0.50	26.90 ± 15.21	-1.36	0.17	-2.40	0.01	-0.44	0.65
	CG	21.49 ± 6.91			21.59 ± 5.90			19.99 ± 7.71			-0.10	0.91	-0.45	0.65
ALT (U/L)	TG	18.41 ± 4.85	-0.96	0.33	15.72 ± 5.01	-0.49	0.62	14.00 ± 5.50	-0.78	0.43	-2.84	0.04	-1.95	**0.05**
	CG	18.73 ± 12.10			18.90 ± 11.43			17.73 ± 9.72			-0.59	0.55	-1.01	0.31
GGT (U/L)	TG	19.93 ± 6.88	-0.58	0.56	18.88 ± 3.90	-0.63	0.52	19.23 ± 5.05	-1.16	0.24	-1.29	0.19	-0.88	0.37
	CG	32.14 ± 30.03			20.29 ± 14.50			39.80 ± 39.82			-1.02	0.30	-2.59	**0.01**
Total proteins (gr/dl)	TG	6.90 ± 0.48	-1.24	0.21	6.90 ± 0.42	-0.06	0.94	7.31 ± 0.42	-0.03	0.97	-1.13	0.25	-2.19	**0.02**
	CG	6.71 ± 0.48			6.88 ± 0.38			7.26 ± 0.34			-1.49	0.13	-2.90	**0.00**
Albumin (gr/dl)	TG	3.93 ± 0.32	-0.09	0.92	3.87 ± 0.18	-0.49	0.62	4.02 ± 0.18	-0.76	0.44	-0.17	0.85	-0.05	0.95
	CG	3.95 ± 0.30			3.82 ± 0.34			4.10 ± 0.26			-0.80	0.42	-1.33	0.18

Note: Between-group (Mann–Whitney *U* test) and within-subject (Wilcoxon’s *T test*) effects for clinical values at three assessment times**.** TG: Treatment Group; CG: Waitlist Control Group; M: Mean ± SD: Standard Deviation); SBP: Systolic Blood Pressure; DBP: Dyastolic Blood Pressure; HR: Heart Rate; MCV: Mean Corpuscular Volume; ESR: Erythrocyte Sedimentation Rate; AST: Aspartate aminotransferase; ALT: Alanine aminotransferase; GGT: Gamma glutamyl transferase; A: pre-treatment assessment; B: post-treatment assessment; C: three-month follow-up assessmen. Bold text: *p* < 0.05.

BMI measures led to a diagnosis of obesity in 6 members of the treatment group and 4 members of the control group (i.e., BMI > 30). Six individuals from the treatment group and 8 controls showed systolic blood pressures greater than 140 mmHg. No participants had diastolic blood pressure over 90 mmHg.

There were no statistical differences between groups in terms of clinical, hematological, and biochemical measures at the beginning of the intervention. The only exception was a greater proportion of polymorphonuclear leukocytes in the treatment group than in the control group (56.4% vs. 48.9%, *p* < 0.005), a difference that is also present at the end of treatment but disappears at follow-up.

After treatment, there were significant differences between groups in the proportion of lymphocytes (TG: 30.66%; CG: 36.99%; *p* < 0.05), although these differences were already present at pre-treatment testing and disappear at follow-up. Three months after treatment, the control group shows a greater proportion of basophils (0.69%) than the treatment group (0.48%) (*p* < 0.05). Also at follow-up, the treatment group showed significantly increased levels of hemoglobin (*p* < 0.02), creatinine (*p* < 0.04), urea (*p* < 0.02), and total protein (*p* < 0.02), as well as decreased levels of the mean corpuscular volume (MCV) (*p* < 0.01) and platelets (*p* < 0.03).

At follow-up, however, the control group also evidenced significant increases in the level of creatinine (*p* < 0.001), HDL cholesterol (*p* < 0.001), GGT (*p* < 0.01) and total protein (*p* < 0.001).

### Immunological testing

Table 4 includes results of the immunological testing for each group at the three assessment times.

**Table 4:** **Immunological measures of both groups at three assessment times**

Variable	Group	A M ± SD	Mann–Whitney*U*		B M ± SD	Mann–Whitney*U*		C M ± SD	Mann–Whitney*U*		Wilcoxon’s*T*A vs. B		Wilcoxon’s*T*A vs. C	
			*z*	*p*		*z*	*p*		*z*	*p*	*z*	*p*	*z*	*p*
CD19	TG	6.60 ± 2.53	-0.23	0.81	4.86 ± 1.33	-1.97	**0.04**	4.72 ± 3.60	-0.80	0.41	-2.43	**0.01**	-1.95	**0.05**
	CG	6.85 ± 1.74			6.75 ± 3.08			5.25 ± 2.49			-0.17	0.86	-1.76	0.07
CD4	TG	41.43 ± 11.40	-0.48	0.62	43.83 ± 6.67	-0.66	0.50	51.90 ± 24.62	-0.06	0.95	-0.66	0.50	-1.51	0.13
	CG	40.36 ± 10.74			45.51 ± 9.15			47.83 ± 10.22			-1.08	0.27	-1.25	0.20
CD8	TG	18.59 ± 6.11	-0.64	0.51	21.13 ± 4.42	-1.28	0.20	22.27 ± 14.24	-0.61	0.53	-1.01	0.31	-0.66	0.50
	CG	18.54 ± 3.51			19.01 ± 6.65			23.08 ± 12.81			0.00	1.00	-0.78	0.43
CD56	TG	20.52 ± 7.72	-0.46	0.64	22.93 ± 5.21	-0.05	0.95	20.18 ± 13.38	-1.05	0.29	-0.87	0.38	-0.53	0.59
	CG	18.58 ± 6.10			24.16 ± 10.93			23.00 ± 7.43			-1.74	0.08	-1.64	0.09
CD71	TG	0.53 ± 0.24	-0.09	0.92	3.52 ± 1.74	-1.98	**0.04**	2.00 ± 1.54	-1.45	0.14	-3.18	**0.00**	-2.94	**0.00**
	CG	0.54 ± 0.30			2.23 ± 1.12			2.25 ± 0.75			-3.11	**0.00**	-3.06	**0.00**
CD97	TG	0.77 ± 0.78	-0.58	0.55	2.90 ± 1.79	-2.46	**0.01**	2.63 ± 1.56	-0.51	0.60	-3.11	**0.00**	-2.58	**0.01**
	CG	0.69 ± 0.57			5.34 ± 2.96			2.33 ± 1.30			-3.18	**0.00**	-2.59	**0.01**
CD134	TG	1.03 ± 0.49	-0.97	0.32	0.28 ± 0.22	-2.26	**0.02**	1.49 ± 0.93	-0.33	0.73	-2.97	**0.00**	-0.48	0.62
	CG	1.26 ± 0.44			0.63 ± 0.44			2.06 ± 2.48			-2.83	**0.00**	-0.40	0.68
CD137	TG	0.14 ± 0.07	-1.09	0.27	0.09 ± 0.07	-2.25	**0.02**	0.63 ± 0.70	-0.25	0.80	-1.73	0.08	-2.14	**0.03**
	CG	0.18 ± 0.10			0.17 ± 0.09			1.19 ± 0.00			0.00	1.00	-1.53	0.12

Note: Between-subject (Mann–Whitney *U* test) and within-subject (Wilcoxon’s *T test*) effects for immunological values at three assessment times. Means are expressed as percentages of total number of lymphoid cells. TG: Treatment Group; CG: Waitlist Control Group; M: Mean (SD: Standard Deviation); A: pre-treatment assessment; B: post-treatment assessment; C: three-month follow-up assessment.

Bold text: *p* < 0.05.

Although there were no significant differences between groups in any of the immunological values prior to treatment, differences in CD19 (Mann–Whitney *U* test; z = -1.97, *p* = 0.04), CD71 (Mann–Whitney *U* test; z = -1.98, *p* = 0.04), CD97 (Mann–Whitney *U* test; z = -2.46, *p* = 0.01), CD134 (Mann–Whitney *U* test; z = -2.26, *p* = 0.02), and CD137 (Mann–Whitney *U* test; z = -2.25, *p* = 0.02), became significant after treatment, only to disappear at the three-month follow-up assessment.

In terms of within-subject effects, at the post-treatment assessment (B) the treatment group showed a significant decrease in CD19+ B lymphocytes (*p* = 0.015), as well as in CD134 lymphocytes (*p* = 0.003), whereas CD71 and CD97 markers were significantly increased (*p* = 0.001 and *p* = 0.002, respectively). Significant changes in CD19 (*p* = 0.05), CD71 (*p* = 0.01), and CD97 (*p* < 0.001) were maintained over the three months following the treatment, while CD137 (*p* = 0.03) values were significantly higher at follow-up.

For the control group, the values of CD71 and CD97 were significantly increased from the pre-treatment to the post-treatment assessment (*p*s < 0.001), and remained unchanged for the three-month follow-up. In contrast, CD134 values were significantly decreased between pre- and post-treatment assessments (*p* < 0.001).

## Discussion

In Spain, this is the first time in which a relaxation technique is used in the treatment of elderly residents of a nursing home and in which psychological, biomedical, and immunological measures are obtained. Our aim was to use this relaxation method as a psycho-gerontological intervention and assess its capacity for enhancing psychological well-being and immune parameters in aging adults. We understand that a “psycho-gerontological intervention” can be construed as a procedure designed to improve the physical and psychological well-being of elderly people by affecting changes at the emotional, cognitive, or behavioral levels. Prior studies have sought to alleviate the immune dysregulation brought about by stress by means of different relaxation techniques (e.g., relaxation or calming responses, progressive muscle relaxation, biofeedback-assisted relaxation) [38–41]. Mahbub-E-Sobhani et al. [42], in their review of immune modulation in response to stress and relaxation, concluded that there is a variety of stressors that have a negative impact on immune function (for instance, by secreting glycocorticoids), but that approaches akin to relaxation techniques could help maintain homeostasis by secreting β-endorphins that trigger an increase in NK cells and, thus, enhance immunity.

Research has shown that immune function is affected both by stress and by normal aging, and these two factors interact, producing a decline in immune activity [18, 43]. The sample in our study was composed of elderly people without obvious stress problems (except for those associated with their health) and without high levels of psychological distress. Nevertheless, relaxation training improved their quality of life and modulated some of their immune parameters. At the psychological level, the relaxation technique produced positive effects in that there was a decline in negative affect, degree of psychological distress, and perception of symptomatology with respect to baseline levels at the beginning of the study. Furthermore, the treatment group showed gains in quality of life at the three-month follow-up.

Our results suggest that the relaxation technique is capable of significantly decreasing psychological distress in a group of elderly people. Research has examined emotional distress and its influence on three important systems: the nervous system, the endocrine system, and the immune system; three systems that interact among themselves and, thus, can disrupt one another [44]. If we take into account that the “Emotion Reactions” scale of the Nottingham Health Profile measures severe psychological distress, and that only the treatment group showed lower scores at the post-treatment assessment, we can conclude that treatment results were clearly beneficial. The NHP measures chronic psychological distress, and not just situational distress. Although the relaxation technique did not enhance positive affect, it was effective in diminishing negative affect, which had been previously linked to disruptions of immune system function [9]. Creswell et al. [16] obtained changes in negative feelings, especially feelings of isolation, by implementing an 8-week Mindfulness-Based Stress Reduction (MBSR) program. In addition to significantly decreasing feelings of loneliness in healthy older adults, the program led to the down regulation of pro-inflammatory NF-κB-related gene expression in circulating leukocytes. Creswell and his collaborators claim that feelings of loneliness diminish because of a change in the perception of social threat that develops by MBSR training [16]. Moreover, they suggest that changes in pro-inflammatory NF-κB-related gene expression are due to the mind-body connection that is generated by the MBSR technique, thus acting on the stress-mediating axes responsible for the expression of the gene. Similar results have been obtained with a group of breast-cancer patients following a psychological intervention [45]. Decreases in feelings of loneliness [16] and those found here in terms of negative affect, are in agreement with the proposal by Quinceno and Vinaccia [23] that, by using relaxation and calming techniques, individuals perceive that there is greater social support, experience feelings of well-being and life satisfaction, obtain better self-awareness and a greater connection with others and with a higher power.

The existence of immune-neuroendocrine communications is widely accepted [46–48]. Those factors that cause changes or alterations in the endocrine system have negative effects on the immune system such as low lymphocyte mitogen response, decreased NK activity, altered T cell populations, or deregulation of cytokines and their receptors [49, 50]. However, the mechanism by which neuroendocrine alterations affect the immune system is unclear [51].

Several parameters have been used to analyze the immune-neuroendocrine relations, in both nonspecific and specific immunity and, within this, both cell and humoral response [52–55].

Our results show significant group differences in various lymphocyte subpopulations after the intervention that disappeared three months later. This could be due to a decrease in practice of the relaxation technique or that this type of treatment shows a greater effectiveness when employed by people under stress associated with immune disorders [56, 57]. However, older people do not always present relevant conditions of acute stress. In fact, stress levels in old age are often well below those found during other phases of the lifespan [58, 59]. Miller and Cohen [10] found that relaxation-based interventions showed little effectiveness in improving stress-related immune disorders because of the insufficient number of trials. Likewise, a higher number of relaxation training sessions may be necessary to produce significant changes in the immunological patterns.

In the elderly, cell marker expression is extremely important due to the decline of the immune system observed in advancing age [18, 60, 61], with a decreased functional capacity observed in studies of responses to mitogen stimulation. The CD4/CD8 ratio is inverted [62] probably due to the decline of the CD4 cells, although some authors describe an increase in this population [63]. Others authors describe a decrease in the number of cytotoxic T cells, although clonal expansions of T CD8+ are also observed [64] and may be due to proliferative responses to tumoral or viral antigens [65]; the mechanisms leading to these changes are still unclear [66].

Whether the increase or decrease of these immune parameters is a marker of clinical or health outcome is not clear. In this study, we describe the changes of B cells (CD19+) population due to that it have been described to decrease in patients with psychological alterations as major depression [67], Alzheimer disease [68] or alcohol dependence syndrome [69] which improved after psychological therapy. This decreasing has also been found in patients with breast cancer having a higher hopeful attitude [70] or in patients that receive psychological treatment before surgery in order to decrease their surgical anxiety [71]. However, B cells are increased in patients with acute schizophrenia [72] or university students under stress periods [73]. In laboratory animals (mouse) with experimental allergic or autoimmune encephalomyelitis [74, 75], as well as in multiple sclerosis patients [76] has also been found the presence of CD134+ cells localized in the active lesions. In children with autism these activation markers decrease significantly after in vitro lymphocyte stimulation [77]. Therefore, the decrease of both cell populations observed in our results after intervention concur with these previous studies and it may be related to the positive effect and improvements observed in these patients. The lack of clarity in the relationship between changes in immune parameters and health consequences might be in part due to different subsets of B cells having different functions and linkage to several systems [1]. However, previous studies [67–77] suggest that people who obtained improvements following an intervention, showed significant changes in the direction indicated in this study. In conclusion, beneficial effects of relaxation techniques on the functioning of the immune system in our study could be inferred.

Our results could be initially explained on the basis of the interconnection of sympathetic system, immune system and psychological well-being, so that the effect of the relaxation training on the sympathetic system is associated with a decline in psychological distress that persists over time, albeit not significantly, and a modulation of immune activity. In addition, our findings on the effect of the relaxation technique on the immune system should be cautiously interpreted, given the following considerations: (a) despite the homogeneity between the two groups (treatment and waitlist control), from the outset there were differences between them in the proportion of segmented leukocytes and lymphocytes, and (b) as shown in Table 4, both groups display significant differences in CD71, CD97, and CD134 markers at the end of treatment. In that sense, our conclusions must be tentative. The immune system of elderly people who show coexisting pathologies and are subjected to multiple pharmacological treatments could be faulty enough to improve on its own.

The present study has limitations that need to be taken into account when considering the study and its findings. Although we have failed to observe consistent immunological changes throughout the study, we did obtain a relative increase in active circulating T lymphocytes. The fading of group differences at the three-month follow-up is reasonable if we take into account that practice of the relaxation technique by the treatment group in the course of those months was inconsistent. Moreover, differences between the pre- and post-treatment assessments in the control group, which obviously could not be due to relaxation, could be attributed to a placebo effect derived from the exhaustive medical testing, psychological assessment, and greater professional support received during the study. Another limitation of this study is the reduced sample size; a larger sample could facilitate the generalization of the results to the Spanish population, as it would permit using more sophisticated statistical methods which provide greater statistical power and to achieve more definitive conclusions. Finally, one aspect to consider, and that could have affected the results of the treatment group, is the potential bias in participant self-reports, given that they could have responded based on what was expected of the treatment. Nevertheless, we consider that this study provides interesting data that clarify our understanding of the effects of the relaxation response on psychological well-being and the immune system in an elderly group residing in a nursing home.

## Conclusions

Through this study we have found that the Benson’s relaxation technique or “tranquilization technique” used here produced an improvement in the quality of life and a modulation of the immune parameters in a group of elderly people residing in a nursing home. Further studies using the tranquilization technique in larger samples of older people are needed to confirm the trends observed in the present study.

Given that it is an easy and economical intervention, it could be useful as a health resource in residential settings where its daily practice could offer medium and long-term benefits for the health and well-being of older adults.

## Authors’ information

ARF is Professor of Health Psychology since 1993, and Head of the Department of Health Psychology (University of Alicante, Spain). His interest topics are psychological therapies in patients with chronic illness and validation of assessment instruments of health-related quality of life. RFC is Associate Professor at the Department of Health Psychology (University of Alicante, Spain). Her research interests concern health-related quality of life and psychological well-being, in different areas and interest groups. ASR is Assistant Professor at the Department of Health Psychology (University of Alicante, Spain). Her research interests concern stress, clinical psychoneuroimmunology and neuropsychology, in both healthy people and patients with chronic disease. ACF is Associate Professor at the Department of Clinical Medicine (University of Miguel Hernandez, Spain) and Head of Section of Immunohaematology at the Blood Bank Centre of Alicante. Her research interests concern immunological alterations in gynaecology, haematology and emotional aspects of Bone marrow transplantation. APS is a geriatrician and Head of the Social Welfare Department (Generalitat Valenciana) in Alicante. His research fields are geriatrics and gerontology as well as integrated care and services for older people. IVR is Associate Professor at the Department of Biotechnology (University of Alicante, Spain). Her research interests concern cell and molecular biology, and clinical immunology in patients with chronic illness. MDFP is Assistant Professor at the Department of Health Psychology (University of Alicante, Spain). Her general research interests are health-related quality of life and well-being in different areas and interest groups. NAB is Assistant Professor at the Department of Health Psychology (University of Alicante, Spain). Her research interests focus on assessment and intervention in the area of mental health.
